# How Denmark, England, Estonia, France, Germany, and the USA Pay for Variable, Specialized and Low Volume Care: A Cross-country Comparison of In-patient Payment Systems

**DOI:** 10.34172/ijhpm.2022.6536

**Published:** 2022-05-07

**Authors:** Wilm Quentin, Victor Stephani, Robert A. Berenson, Lone Bilde, Katja Grasic, Riina Sikkut, Mariama Touré, Alexander Geissler

**Affiliations:** ^1^Department of Health Care Management, Technische Universität Berlin, Berlin, Germany.; ^2^European Observatory on Health Systems and Policies, Brussels, Belgium.; ^3^HelloBetter, Berlin, Germany.; ^4^The Urban Institute, Health Policy Center, Washington, DC, USA.; ^5^Danish Institute for Applied Social Sciences Research, Copenhagen, Denmark.; ^6^Danish Cancer Society Research Centre, Copenhagen, Denmark.; ^7^Centre for Health Economics, University of York, York, UK.; ^8^The Estonian Parliament, Tallinn, Estonia.; ^9^Poverty, Health and Nutrition Division (PHND), International Food Policy Research Institute (IFPRI), Washington, DC, USA.; ^10^School of Medicine, University of St. Gallen, St. Gallen, Switzerland.

**Keywords:** Prospective Payment System, Reimbursement Mechanisms, Healthcare Financing, International Comparison, Tertiary Healthcare

## Abstract

**Background:** Diagnosis-related group (DRG)-based hospital payment can potentially be inadequately low (or high) for highly variable, highly specialized, and/or low volume care. DRG-based payment can be combined with other payment mechanisms to avoid unintended consequences of inadequate payment. The aim of this study was to analyze these other payment mechanisms for acute inpatient care across six countries (Germany, Denmark, England, Estonia, France, the United States [Medicare]).

**Methods:** Information was collected about elements excluded from DRG-based payment, the rationale for exclusions, and payment mechanisms complementing DRG-based payment. A conceptual framework was developed to systematically describe, visualise and compare payment mechanisms across countries.

**Results:** Results show that the complexity of exclusion mechanisms and associated additional payment components differ across countries. England and Germany use many different additional mechanisms, while there are only few exceptions from DRG-based payment in the Medicare program in the United States. Certain areas of care are almost always excluded (eg, certain areas of cancer care or specialized pediatrics). Denmark and England use exclusion mechanisms to steer service provision for highly complex patients to specialized providers.

**Conclusion:** Implications for researchers and policy-makers include: (1) certain areas of care might be better excluded from DRG-based payment; (2) exclusions may be used to incentivize the concentration of highly specialized care at specialized institutions (as in Denmark or England); (3) researchers may apply our analytical framework to better understand the specific design features of DRG-based payment systems.

## Background

**Key Messages**

** Implications for policy makers**

- Certain areas of care are excluded from diagnosis-related group (DRG)-based payment in reviewed countries.
- Complexity of exclusion mechanisms and associated additional payments differ across countries.
- Exclusion mechanisms can help steer service provision for highly complex patients.
- Our analytical framework facilitates understanding specific design features of DRG-based payment.

** Implications for the public**

Hospitals in most high-income countries are paid based on diagnosis-related groups (DRGs). DRG-based payment systems assign patients into groups that are medically meaningful and have similar costs, and payment is determined based on the numbers and types of DRGs provided. However, certain patients with rare conditions or requiring special services may not be adequately reflected by DRG-based payment, as payment for these patients could be inadequate, ie, either too high or too low. Our study reviews approaches used in six countries (Germany, Denmark, England, Estonia, France, the United States) to overcome this problem. We find that mechanisms used to supplement DRG-based payment differ across countries but that similar areas of care, eg, cancer care and specialized pediatrics, are often excluded from DRG-based payment. Policy-makers introducing or reforming DRG-based payment may benefit from looking at mechanisms used to supplement DRG-based payment systems in other countries, ultimately contributing to better quality and accessibility of care.

Internationally, diagnosis-related group (DRG)-based payment systems have become the main mechanism for the reimbursement of acute inpatient care.^1,2^ DRG systems classify hospital cases based on the diagnoses and procedures of a patient into a manageable number of clinically meaningful and economically homogeneous groups.^3^ Each DRG should ideally contain cases that have comparable costs in order to allow for reliable calculation of average costs per DRG.

 However, all DRG systems struggle with the problem that average costs are difficult to calculate for certain groups of patients. This is because some DRGs group together patients with highly variable costs, while other DRGs, in particular those for relatively specific and complex diseases, contain only relatively few cases.^4^ This problem is exacerbated by that fact that more complex patients (eg, those with multiple comorbidities) tend to have costs that are more variable, potentially leading to more high-cost outliers.^5^ As patients with highly variable costs are often treated by highly specialized (tertiary) hospitals, these providers may face financial difficulties if DRG-based payments (based on average costs) are too low for these specific patients.^6-9^ In addition, high variability of costs can be problematic for high-cost patients, as it may lead to unintended consequences, such as cream-skimming, dumping, undertreatment, or inappropriate early discharges.^7,10^ At the same time, given that these high-cost patients often consume a sizeable share of total hospital costs, average costs of DRGs would be too high for average patients treated in most hospitals if the costs of these high-cost patients or of certain high-cost services were not excluded during calculation of average costs.^11^

 In order to better reflect resource consumption of hospitals, DRG-based payment systems have been supplemented in many countries by excluding certain elements from DRG-based payment, which are then reimbursed through additional payment mechanisms. The most common exclusion mechanism used in almost all countries with DRG-based payment, is the use of outlier payment adjustment.^10,11^ Typically, long-stay outliers are identified using a certain threshold, eg, average length of stay (LOS) plus twice the standard deviation of LOS. The costs of cases staying beyond this threshold are excluded when calculating average costs of DRGs, and hospitals are reimbursed with a per diem payment for the additional days that patients stay in hospitals beyond this threshold. Furthermore, several countries have short-stay outlier adjustments, where hospitals do not receive the full DRG-based payment if the LOS is below a specified short-stay threshold.

 Other payment adjustments include various forms of additional budgets, fee-for-service (FFS) payments, or per diems. All these mechanisms have in common that certain elements of inpatient care are excluded, when calculating DRG-based payment, and subsequently they are paid for separately. One example are certain high-cost services, such as dialyses, which are provided to patients classified into various DRGs but not to all patients classified into these DRGs.^1^ As a result, dialyses would increase variability of treatment costs of patients within these DRGs. Therefore, in order to improve homogeneity of treatment costs, dialyses are usually excluded from calculating average costs of DRGs, and they are reimbursed separately through FFS. Another example are patients with severe burns whose costs are highly variable because some patients require very long stays in hospitals.^12^ Because of relatively low case numbers and high variability of costs, it is difficult to reliably calculate average costs of burns patients. Therefore, burns patients are often excluded from DRG-based payments and hospitals are reimbursed through alternative payment mechanisms, eg, negotiated budgets or case-payments.

 As no systematic overview of these additional payment mechanisms is available, the aim of this paper is to compare payment mechanisms that complement DRG-based payment across countries. More specifically, the objectives were (1) to develop a conceptual framework for the analysis of exclusion mechanisms and additional payment components; (2) to use the framework for the analysis of exclusion mechanisms and additional payment components in six countries; and (3) to identify specific areas of care for which acute care hospitals in different countries receive additional payment components.

 The focus is on mechanisms that attempt to reduce variability or target particular areas of highly specialized or low-volume care. Given that outlier payments are common to all DRG-based payment systems and that the specific mechanisms for calculating different thresholds and reimbursement levels for length-of-stay or cost-outliers have been described previously, we do not focus on differences in outlier payments across countries.^10,11^ In addition, payment mechanisms that primarily aim at reimbursing the costs of new diagnostic and treatment methods are not further considered in this paper as specific studies have already provided an overview of these mechanisms.^13-15^ Moreover, psychiatric, rehabilitation, and long-term care hospitals, which are often excluded from DRG-based payment systems,^16^ are outside the scope of this paper. Furthermore, it is important to note that this paper focuses on the regular payment mechanisms of hospitals, which have been altered in several countries as a result of coronavirus disease 2019 (COVID-19).^17^ For example, the DRG-like hospital payment system in England has been replaced with global budgets and Germany introduced per-diem payments for empty beds.

## Methods

###  Conceptual Framework

**Figure 1:**
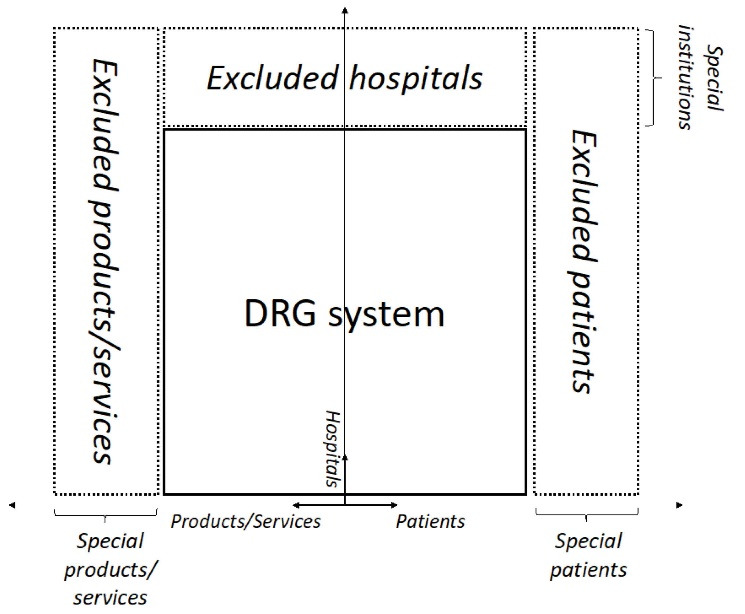
Framework Used for Comparative Analysis. Abbreviation: DRG, diagnosis-related group.

 Based on previous research,^13,18-22^ and theoretical considerations,^23,24^ three main mechanisms were identified that attempt to reduce variability of costs within DRGs: (1) the exclusion of certain patient groups (eg, patients with severe burns, palliative patients), (2) the exclusion of certain services and products (eg, high-cost drugs, devices, intensive care), and (3) the exclusion of certain hospitals or hospital departments (eg, highly specialized departments/hospitals, such as epilepsy departments, cancer hospitals). Subsequently, we developed a conceptual framework to guide our cross-country analysis of payment mechanisms that complement standard DRG-based payments (see Figure 1). The figure illustrates the idea that there is a core DRG system, which includes most patients, services and hospitals. Moving along the arrows from the centre of the core DRG system to the periphery, patients, services, and hospitals are becoming increasingly more complex or specialized. At some point, patients, services, and hospitals have reached a point on the continuum, where they are so ‘special’ that they are excluded from DRG-based payment because they are no longer considered to fit into the core DRG system. However, it depends on the specific country, when this point is reached and what is considered to be ‘special.’

 Payment components for special services/products are shown on the left-hand side of the figure (eg, high cost drugs). If a particular service or product is excluded from the calculation of DRG-based payment, this means that the DRG implicitly pays for all other services provided during the inpatient stay, and only the excluded services are reimbursed separately. Payment components for special patients are shown on the right hand of the figure (eg, DRGs for patients with severe burns that do not have cost-weights). If certain patients are excluded, all services provided to these patients during a hospital stay are excluded from DRG-based payment. Payment components for special hospitals/departments, are shown at the top (eg, specialized hospitals). If the entire hospital is excluded, payment for all patients treated by the hospital is separate from the DRG-based payment system.

###  Country Selection

 Based on a rapid review of European Observatory Health System in Transition reviews, previous research,^1,13,19,25,26^ and authors’ experience, a long list of countries was drafted, where DRG-based payments for hospitals are supplemented by other payment mechanisms for specific patient groups, hospital stays or services/products. Table S1 in Supplementary file 1 provides an overview to the countries that were included on the long list. For countries on the list, information was collected on the basic characteristics of the DRG system, the use of additional payment mechanisms, the availability of established contacts with DRG-experts, and other aspects such as interesting developments or recent reforms. Based on these criteria, countries were assessed and six countries were included in the final analysis: Denmark, England, Estonia, France, Germany, the United States (Medicare). In the United States, the Medicare system, which mostly covers people aged 65 and older, was included as this is the largest public insurance program, with a DRG-based hospital payment system that is some similar to that in European countries.^19^ Table S1 explains the reasons for exclusion. These included, for example, if the payment system was characterized by a wide degree of in-country variation and/or that reimbursement rules were determined in a non-transparent negotiation process.

###  Data Collection

 Information on DRG-based payment systems is often not described in sufficient detail in the available literature. Therefore, a questionnaire was developed to obtain comprehensive and detailed information for the selected countries from national researchers (co-authors of this paper). The questionnaire was based on the framework and consisted of three sections (see Supplementary file 2): The first section focused on background information about the national DRG-based payment system, asking for information on the proportion of total hospital revenues related to DRG-based payment as well as the process of developing and updating the DRG system. The second section focused on the different exclusions from DRG-based payment, with subsections for (*a*) excluded patient groups, (*b*) excluded services and products, (*c*) excluded hospitals or departments, (*d*) outliers, and (*e*) other mechanisms. Each of these subsections asked for information on the specifically excluded patients/services/departments, the process for making decisions on exclusions, and the mechanisms used to pay for these patients/services/departments. Finally, a third section focused on main challenges, debates, and reforms related to the problem of high variability of costs.

 National researchers from the included countries (co-authors of this paper) reviewed relevant national statistics, policy documents, and available literature and provided written answers to the questions by mid-2017. They also commented on discrepancies between formal regulations and payment in practice, eg, concerning the relevance of ‘local variations’ in England. The completed questionnaires were then reviewed and validated by the coordinating research team. Technical reports and studies mentioned by national researchers were cross-checked and complemented by further literature searches (grey and peer-reviewed). Subsequently, national researchers answered additional questions about points that had remained unclear in their original response. Remaining ambiguities were iteratively clarified through further correspondence. This led to a detailed assessment of each country’s DRG system. Prior to submission of the paper in April to July 2020, national researchers reviewed the draft manuscript and updated text and tables if necessary.

## Results

 DRG-based hospital payment systems in the six included countries differ with regard to several key characteristics (see Table 1), which influence both the need of these systems to accurately reflect treatment costs and their ability to do so. First, accurate reimbursement of treatment costs is more important when a large proportion of hospital costs is reimbursed by DRGs. While numbers are difficult to compare across countries because of regulatory and organizational differences, the proportion of DRG-based payment of total hospital costs varies roughly between 60% and 80%.

**Table 1:** Range of Costs Included in the DRG-Based Payment Systems

	**DRG-Based Payment in Combination With**	**Number of DRGs**	**Range of Costs Included in DRG-Based Reimbursement**	**Outliers Based on**
Denmark	DRGs (≈80%), budget (≈20%)	743 (in 2017)	The payment covers all hospital costs except education & research, depreciation and capital costs	LOS
England	DRGs (≈63%), budgets	2516	Tariff includes all operating expenses, staff costs and capital costs (both interest and principal), but excludes the costs of education & research	LOS
Estonia	DRGs (70%), FFS (30%)	800	The payment covers all hospital costs except education & research	Cost (high and low-cost outliers)
France	DRGs (≈63%), budgets, FFS (in private hospitals)	2300	All costs are covered except for education & research costs and payments for physician fees in private, for-profit hospitals	LOS
Germany	DRGs (≈71%), budgets, FFS	1255	All costs except costs for investing in/maintaining infrastructure and education & research	LOS
USA	DRGs (≈80%), FFS (from other payers)	756	All costs except fees for physicians and some education & research	Cost (only high-cost outliers)

Abbreviations: DRG, diagnosis-related group; FFS: fee-for-service; LOS, length of stay.

 In Denmark, the regions pay for hospital care using DRG-based payments that explicitly cover 80% of costs of every patient, while negotiated budgets cover the remaining 20%. In Estonia, DRG-based payments from the Estonian Health Insurance Fund explicitly cover only 70% of patient costs, while FFS covers the remaining 30%. In France, and Germany, DRG-based payment from sickness funds, in principle, cover average treatment costs of DRG cases but hospital payment includes other payment components (budgets, FFS). The same is true also for healthcare resource group (HRG)-based hospital payments from the National Health Service (NHS) in England. In the USA, DRG-based payment makes up almost 80% of hospital payments from Medicare but hospitals have multiple other revenue sources from public (eg, Medicaid) and private (eg, employer-sponsored insurance) payers. In addition, physician fees are excluded from DRG-based payment in the United States under the Medicare system, while they are included in the other countries (except in private hospitals in France). Out-of-pocket payments do not play an important role in financing hospital care in any of the included countries, as they account for less than 2% of hospital expenditures in England, France, and Germany, reaching 4.4% only in Estonia.

 Secondly, DRG systems with a larger number of DRGs should – at least in theory – be able to better reflect actual treatment costs due to their higher granularity. Germany, England and France have a relatively high number of DRGs, mostly related to a higher number of severity levels per DRG (in Germany this is almost unlimited). In Denmark, Estonia and the United States there are fewer groups and also fewer severity levels. However, existing research shows that DRG systems with a higher number of groups are not necessarily better at predicting costs of care as the specific classification variables and algorithms that determine the definition of DRGs in DRG-based payment systems are important.^1,20^ For example, some DRG systems classify patients with stroke into different groups depending on whether or not they are treated on a stroke unit and whether or not they receive systemic thrombolysis, while other countries do not consider these variables in their classification algorithms.^21^

 Thirdly, the system for outlier reimbursement, ie, payments for cases that exceed or fall below a certain limit in terms of costs and/or LOS, may influence the need for additional payment component besides DRGs. A more precise system can reduce variability of costs for DRG inliers. In Estonia and the United States, the reimbursement for outliers is more accurate as high-cost outliers are defined based on costs. In other countries, outliers are defined by LOS.

###  Overview of Elements Excluded From DRG-Based Payment

**Figure 2:**
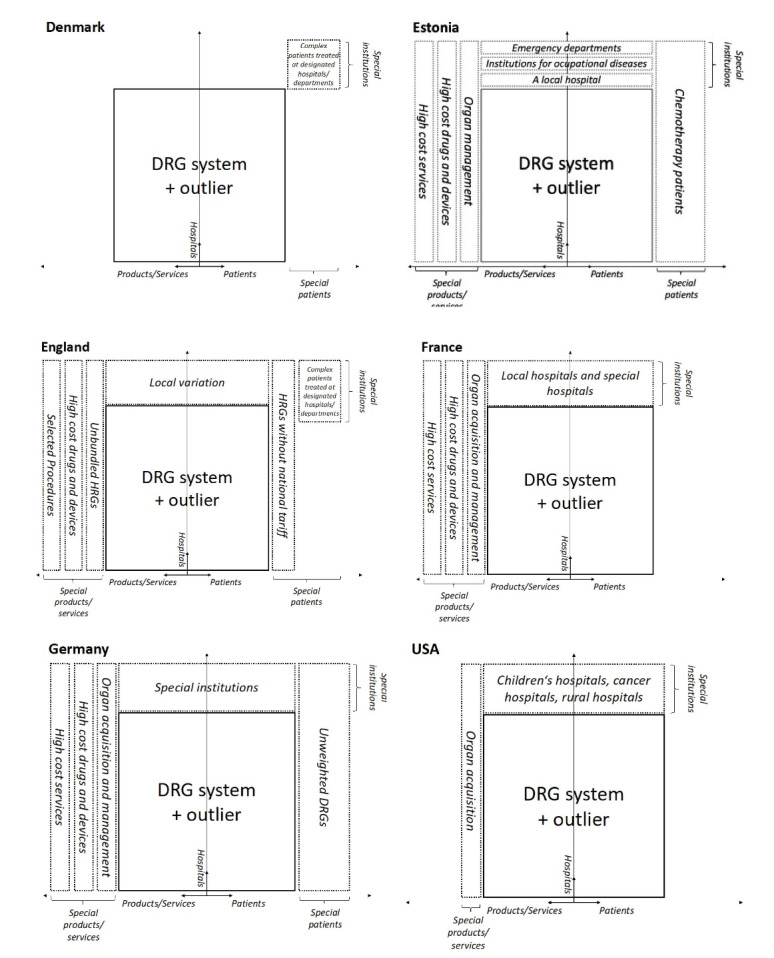
Overview of Elements That Are Excluded From DRG-Based Payment in Denmark, Estonia, England, France, Germany, and the United States. Abbreviation: DRG, diagnosis-related group.

Figure 2 provides an overview of the elements excluded from DRG-based payment in the six included countries. The figure shows that all countries exclude certain patients, certain services, and products, and/or certain hospitals and/or departments from DRG-based payment. These mechanisms can also be combined at the institutional level. For example, excluded hospitals and/or departments may receive additional reimbursement for excluded services and or patients.

 In *England, Estonia, *and* Germany *all three exclusion mechanisms are applied: certain patient groups, certain services/products and certain hospitals/departments are excluded from DRG-based hospital payment. In*France* several services, high-cost drugs and hospitals are excluded from DRG-based payments, but no patient groups. The *US* Medicare (Part A) reimbursement system makes only relatively few exceptions from the DRG-based payment: certain hospitals are excluded and the service of the acquisition of organs is reimbursed separately.

 In *Denmark*, an approach that combines two criteria is used: highly complex patients are excluded from the DRG-based payment system – but only if these patients are treated at designated hospitals/departments. A similar approach exists also in *England*, where top-up payments are provided for some specialized services if they are provided at certified departments.

###  Payment for Excluded Elements

 In *Denmark*, approximately 10% of all acute inpatient cases are excluded from the DRG based payment system.^27^ Locally referred to as ‘complex patients,’ these cases receive either *specialized* or *highly specialized services, which* can be offered only by designated providers, so-called *special functions (departments)*. Currently there are around 1000 specialized services (including highly specialized ones) across 36 medical specialties (eg, transplantations or fetal surgeries). (Highly) Specialized services are defined as services that are highly complex, rare or particularly resource intensive. Hospitals have to apply to the Danish Health Authority in order to be eligible to provide these services. The Danish Health Authority will review applications and designate institutions to perform the service after consideration of available medical expertise and resources as well as population health needs. In terms of payment, each specialized department which undertakes these services receives a pre-payment by the region, which accounts for 25% of last years’ total payment for complex patients. Payment for each patient is settled later, eg, at the end of the year, based on retrospective reimbursement of costs as calculated by the individual hospital.

 In *England*, there are 341 HRGs which do not have a national tariff (eg, patients receiving haemodialysis, transplantation or having severe burns). Tariffs for these HRGs are locally negotiated. Additionally, there are 107 HRGs with non-mandatory tariffs (including 50 HRGs covering the maternity pathway), which are used as the basis for local negotiations between the provider and the Clinical Commissioning Group (CCG) and as an aid for provider-to-provider charging. Furthermore, several high-cost drugs, devices, and diagnostic services are excluded from core-HRGs and generate so-called unbundled HRGs. Unbundled HRGs can be considered FFS payments. However, out of 277 unbundled HRGs, 157 do not have a national price and are locally negotiated. So, in addition to the 341 HRGs without a national tariff mentioned above, there are also 277 unbundled HRGs without a national tariff. In addition, hospitals can be excluded from the HRG based payment system and reimbursed based on a negotiated budget (block contract) if they have a special arrangement with their CCG (known as ‘local variations’). The number of local variations has increased significantly over the last years, but it is not known how many hospitals operate under such local contract agreements.

 For all components without a national tariff, local tariffs are negotiated between CCGs and providers. In fact, CCGs have considerable flexibility with regard to defining the payment modalities. This contributes to large variation in the way the local prices or budgets are set. Furthermore, local arrangements can be made for HRGs with national tariffs, in cases where the set tariff does not adequately reimburse the costs due to certain structural, local circumstances.

 Finally, somewhat similar to the Danish approach, the NHS pays a top-up for certain patients (HRGs) with complex healthcare needs who are treated in designated departments. These top-up payments apply to certain services in the pediatrics, cardiac and pulmonary departments, neurology, spine surgery and orthopedics departments.^28^ They also apply for selected cancer treatments.

 In *Estonia*, the only patient group excluded from the DRG-based payment system is certain patients receiving chemotherapy sessions. Besides, several high-cost drugs, devices and services are excluded (eg, therapy with biologicals for Multiple Sclerosis or hearing implants). In addition, tuberculosis-departments and departments/‘beds-reserved’ for occupational diseases are separately reimbursed. Elements that are excluded from DRG-based payment in Estonia are reimbursed using a combination of per diems and FFS payments.

 In *France*, high-cost drugs and devices are excluded and paid separately based on a nation-wide fee catalogue. In addition, certain services which can be added to a core DRG in case particular conditions are met (eg, malfunctioning of an organ, artificial respiration, intensive care patient) are excluded from the DRG-based payment system and mostly reimbursed based on per diems. Hospitals also receive additional fee-per-session payments for dialysis patients without chronic kidney insufficiency, and they are eligible to receive block grants for the coordination and management of organ transplantations. Furthermore, local and small-scale hospitals (8.4% of all hospitals) are paid by a mixture of block grants (based on historic costs), regional characteristics and the activity produced.^29^

 In *Germany* there is a list of patient groups (DRGs) that do not have a cost weight. In 2017, the list included 45 *unweighted DRGs* (eg, bone marrow transplant patients and tuberculosis patients). Furthermore 192 products/services (eg, hemodialysis or hemoperfusion) and 96 pharmaceuticals (or 1538 with various dosage forms) are excluded from the DRG-based payment system, as well as the management (and transportation and removal) of organ transplantations.

 Another exclusion mechanism is the exclusion of certain hospitals or hospital departments, which are classified as *special institutions*. Special institutions are defined as departments/hospitals with a focus on the following specialties: palliative care (with a minimum of 5 beds), child and youth-rheumatology, tropical diseases, multiple sclerosis, morbus Parkinson and Epilepsy. Furthermore, certain children’s hospitals and low-volume departments that are essential from a societal perspective (eg, isolation wards) are also excluded. Finally, hospitals can be excluded if three quarters of all cases have a LOS above the average. Excluded hospitals are usually paid on the basis of per diems.

 DRG-tariffs for unweighted DRGs are negotiated at the hospital level, while excluded services/products are paid for with an FFS (either nationwide-tariff or negotiated at hospital level).

 In the *US Medicare system*, no patient groups are excluded from the DRG-based payment system. The only product/service excluded is the cost of organ acquisition for transplant cases.^30^ Apart from that, rural hospitals (so-called critical access hospitals) and certain cancer-hospitals/departments are separately reimbursed. Children’s hospitals are also excluded because Medicare mostly covers people aged 65 and older, which means that the DRG-based payment system was not developed to account for the costs of care provided to children. Excluded hospitals are paid on the basis of their incurred costs. Organ acquisition of transplant cases is also reimbursed based on each (certified) center’s incurred costs. Table 2 provides more details on the excluded elements.

**Table 2:** Detailed Overview of Exclusion Mechanisms Used in Denmark, England, Estonia, France, Germany, and the United States

**Country**	**Exclusion Mechanism **			
	**Patient Groups**	**Products/Services**	**Departments/Hospitals**	**Other**
Denmark	-	-	-	Cost based reimbursement for ‘Complex patients,’ ie, those receiving specialised services (n = 1000), treated in designated specialised departments
England	Negotiated HRG-based payments for 341 out of 2 516 HRGs	Negotiated FFS payments for high-cost drugs, devices and selected procedures and diagnostic imaging	Decentralised system: the exclusion of hospitals depends on the local CCG (payments based on local negotiation)	Top up payments for specialised departments providing ‘highly specialised services’ to patients
Estonia	Payments based on a combination of per diems and FFS for chemotherapy patients	Payments based on a combination of per diems and FFS for high-cost drugs, devices, services, organ transplantation	Payments based on a combination of per diems and FFS for departments for occupational disease/tuberculosis	-
France	-	Block grant for organ management/harvesting/transplantation; mostly FFS-based payments for high-cost drugs (n = 518), devices (n = 58): mostly per diem based payments for services (n = 20)	Payment based on a mixture of block grants and activity for local hospitals/ special institutions (n = 164, 8.4% of all acute care hospitals in 2015)	-
Germany	Negotiated DRG based payments for 45 out of 1 255 DRGs (in 13 major diagnostic categories)	Cost based payments for organ management/harvesting/ transplantation; nationwide or negotiated tariff-based payments for high-cost drugs, devices, services (total n = 191)	Per diem based payments for special institutions (n = 153 in 2016)	-
USA (Medicare)	-	Cost based payments for organ acquisition for transplant cases	Cost based payments for children’s hospitals (n = 11)/cancer hospitals (n = 60)/ hospitals in Maryland/Critical access hospitals (small, rural hospitals; n = 1300)	-

Abbreviations: DRG, diagnosis-related group; FFS: fee-for-service; HRG, healthcare resource group; CCG, Clinical Commissioning Group.

###  Mechanisms Used for Particular Areas of Care

Table 3 shows examples of national approaches for dealing with certain medical fields or treatment areas in order to pay for variable, specialized and low volume care. Some areas of care, such as organ transplantations and certain cancer treatments are excluded from *all* DRG systems, while many other elements, such as specialized paediatric or dialysis services, are excluded from *most* DRG systems. And yet other elements are only excluded from relatively *few* DRG systems, such as intensive care or severe burns.

**Table 3:** Clinical Areas Excluded From DRG-Based Payment in Denmark, England, Estonia, France, Germany, and the United States

**Area of Care**	**Exclusion Mechanisms Used**			
	**Patient Groups**	**Services/Products**	**Hospitals/Departments**	**Other**
Oncology	England (bone marrow transplantation), Estonia (chemotherapy), Germany (bone marrow transplantation)	Estonia, England, France, Germany (costly cancer drugs)	USA (certain cancer hospitals)	Denmark (eg, treatment of pancreatic cancer)
Specialised paediatrics	Germany (neuro-paediatrics), England (paediatric intensive care)	France (eg, paediatric intensive care), Germany (neuro-paediatric diagnostics)	Germany (eg, child-rheumatology), USA (60 children hospitals)	Denmark (eg, paediatric intensive care), England (several specialized services)
Severe burns	England, Germany (severe burns)	-	Germany (severe burns)	Denmark (severe burns)
Neurology	Germany (eg, multimodal, complex treatment against Parkinson)	Estonia (biologic therapy against multiple sclerosis)	Germany (eg, multiple sclerosis)	England (several specialized services)
Dialysis	England (eg, hospital haemodialysis or filtration)	France, Germany (dialysis)	-	Denmark (eg, peritoneal dialysis), England (insertion and removal of the peritoneal dialysis catheter for children)

Abbreviation: DRG, diagnosis-related group.

####  Cancer Treatment

 All analyzed countries exclude certain elements of cancer treatment from DRG-based payment. In *England, France *and* Germany* an increasing number of oncological drugs are excluded and reimbursed FFS. In *Estonia, England *and* Germany*, some patient groups (eg, chemotherapy patients in Estonia, and bone marrow transplantation patients in England and Germany) are excluded and reimbursed through negotiated prices or a combination of FFS and per diems (Estonia). In the *United States* (Medicare) selected cancer hospitals are completely excluded from the DRG-based payment system and reimbursed on the basis of “reasonable costs.” In *Denmark,*complexcancer patients who need highly specialized treatment services (eg, for pancreas or kidney cancer) are excluded from DRG-based payment if provided at designated hospitals.

####  Specialized Pediatrics

 All countries except Estonia have exceptions from DRG-based payment for certain pediatric cases. Some pediatric DRGs in *Germany *and* England* do not have national prices but are reimbursed based on negotiated prices. In England, this includes pediatric cystic fibrosis and developmental issues. Furthermore, children’s hospitals and certain special institutions(eg, for child- and youth-rheumatology) are excluded from DRG based payment in Germany and mostly paid based on per diems. Also, under the Medicare program in the *United States*, 60 children’s hospitals are excluded from DRG-based payment and reimbursed on the basis of reasonable costs. In *France* supplementary per diem payments exist for certain pediatric services, ie, for pediatric intensive care and for neonatal care. Similarly, neuropediatric diagnostic services are paid FFS in *Germany*. In *Denmark*, several highly specialized pediatric patients are excluded from DRG-based payment if treated by designated providers. Also in England, designated specialized institutions receive top-up payments for treating complex pediatric patients (for 13 selected conditions). Top up payments range from an additional 11.93% for special gastroenterology and hepatology services up to an additional 79.27% for pediatric cancer services.

####  Severe Burns

 Provisions for the exclusion of severe burns from DRG-based hospital payment exist in Germany, Denmark, and England. Hospitals or departments for severe burns that have been deemed necessary by needs assessment can be excluded from the *German* DRG system, which also includes a few unweighted DRGs for severe burn cases. In *Denmark* designated hospitals for the treatment of severe burns are reimbursed on the basis of their own calculated costs. Care for patients having severe burns is paid for with locally negotiated tariffs in *England*. The commissioners select the reimbursement method and can choose to incorporate different models, ie, integrated care tariffs or paying FFSs.

####  Neurology

 Certain elements of neurology are excluded from DRG-based payment in Germany, Estonia, and England. In *Germany* hospitals for the treatment of certain neurological disorders like multiple sclerosis or epilepsy are excluded from DRG-based payment, and reimbursed based on locally negotiated case-based payments or per diems. In *Estonia*, therapy with biologicals for multiple sclerosis is reimbursed based on a combination of per diems and FFS. In *England*, top-up payments (ranging from 7.54%-35.52%) for complex patients in certified (highly) specialized areas are granted for interventions in the field of neurology.

####  Dialysis

 In *Denmark*, dialysis treatments are excluded from DRG-based payment and reimbursed based on the treating hospitals’ own cost calculation. For dialysis services in *France*, hospitals receive a supplementary fee per session, called ‘dialysis package,’ in addition to a standard DRG-based payment. Similarly, several supplementary fees exist for different dialysis-services (eg, hemodialysis, peritoneal dialysis) in *Germany*. In *England*, HRGs for ‘hospital haemodialysis or filtration,’ ‘home haemodialysis’ and ‘ambulatory peritoneal dialysis’ have no national price. Haemodialysis and peritoneal dialysis for acute kidney injury are unbundled. Most of the unbundled services and HRGs with no national tariff are reimbursed based on locally negotiated tariffs. Furthermore, designated hospitals receive a top-up payment if they insert/remove a peritoneal dialysis catheter for children.

## Discussion

 All countries with DRG-based payment systems struggle with the problem that average costs cannot be reliably calculated for DRGs in certain areas of care. Certain patients, services, and/or hospitals are therefore often excluded from DRG-based payment. This cross-country comparison provides the first systematic analysis of these exclusions and of the related additional payment mechanisms that complement DRG-based payment systems in six high-income countries. In summary, there are three main findings of this paper: First, our overview shows that the complexity of exclusion mechanisms and additional payment components differs across countries. While some countries, such as England and Germany use many different exclusion mechanisms and have a high number of additional payments, other systems, such as Denmark or the United States (Medicare), have significantly fewer exceptions. Second, despite these differences, most countries exclude similar areas of care from DRG-based payment systems. This almost always includes certain areas of cancer care and specialized pediatrics. Third, while the primary rationale of exclusion mechanisms is to reduce variability within DRGs, Denmark and England, use exclusion mechanisms to steer service provision for complex patients to highly specialised providers.

 These findings have important implications for researchers and policy-makers. First, the differences in exclusion mechanisms across countries underline the need for researchers and policy-makers to carefully assess the specific details of different countries’ hospital payment systems in order to understand their differential effects on efficiency and quality.^31^ Intended and unintended effects of DRG-based payment systems are strongly influenced by the specific design features of these systems.^10^ Previous empirical studies about the effects of DRG-based payment systems on efficiency and quality of care have found inconsistent results across countries, with some studies showing increased activity and mortality while others found no effects on volumes or quality of care. One possible explanation for inconsistent results is that the additional payment components complementing DRG-based payment systems moderate the overall effects of these systems. For example, while DRG-based payment generally has incentives to reduce the number of services provided per case,^10^ additional FFS-based payments for excluded services would reduce this incentive. However, it is important that differences in additional payment components are considered together with the specific design features of the general DRG-based payment system: For example, in the United States (Medicare) and in Estonia, outlier payments are determined based on costs (see Table 1), which enables a more accurate payment adjustment than outlier payments based on LOS. In addition, other aspects have to be considered as well, eg, the number of DRGs and the proportion of total hospital costs financed through DRGs. Therefore, it is important for policy-makers to consider the specifics of implementation when developing or reforming DRG-based payment systems, and for researchers to avoid simplistic assumptions about the effects of DRG-based payment systems when making quantitative cross-country comparisons.^35^

 Second, our results may provide guidance to policy-makers introducing or reforming DRG-based payment systems as they will have to make decisions about the appropriate scope of the system, ie, which services, patients and facilities should be included or excluded. Previous research has discussed the trade-offs between providing top-up payments to hospitals, ie, increasing the DRG-price for certain providers, and the possibility of refining DRG systems by splitting existing DRGs into less and more complex groups.^5,36^ Refinement is most appropriate when patients that receive complex care are concentrated in a small number of DRGs that are provided by many hospitals. If patients are spread across many DRGs, subdividing them will generate many more DRGs, containing fewer patients thus complicating reliable calculation of average costs. Furthermore, if these patients are treated by only one or two hospitals, the price of the DRGs will directly reflect the costs of care. In such cases, Bojke et al argue that a top-up payment would be preferrable^5^ and they discuss statistical methods for identifying those DRGs that should be refined or receive a top-up. Given that exclusion mechanisms in almost all countries target organ transplantations or certain elements of cancer care, specialized paediatrics and neurological care (see Table 3), it seems that these areas of care are difficult to include in DRG-based payment systems. Therefore, policy-makers may benefit from looking at our results – and at the different country specific exclusion lists – to better understand how other countries define exclusion mechanisms for these areas of care and how they reimburse excluded elements. While our results do not allow to identify the most suitable reimbursement mechanisms for excluded elements, it is clear that most countries use FFS payments for excluded services or products. More variation exists concerning excluded patient groups or excluded hospitals/departments, which are reimbursed using different combinations of budgets, negotiated case-based payments, FFS, or cost-based reimbursement. Future research could attempt to assess the effects of different exclusion mechanisms and additional payment components on efficiency and quality of care.

 Third, it is important that policy-makers are aware of the incentives created by different exclusions and payment mechanisms. Excluding a large number of services and high-cost drugs from DRG-based payment and reimbursing these using FFS creates explicit incentives for the provision of these services.^24,37^ However, while this may assure access to these services for affected patients, it also reduces incentives for efficient use of resources. Similarly, allowing cost-based reimbursement for excluded patient groups (as is the case in Denmark), eliminates any incentives for efficient use of resources, when treating these patients. In the United States, in order to limit financial risk for the payer, the Centres for Medicare and Medicaid Services has so far refused to create additional FFS payments for specific products, when the product in question is a sole-source product under patent and the manufacturer has monopoly power. This explains why the number of excluded services is low in the United States. However, such an approach is probably possible only in a context, where outlier payments are based on costs.

 Last, many countries in Europe struggle to concentrate service provision for highly specialized care at fewer providers in order to improve both efficiency and quality of care.^5,38,39^ In this context, our results provide an interesting example about how to support the concentration of highly specialized care through the payment system. In Denmark and England, exclusion from DRG-based payment is used to steer service provision for highly complex patients to specialized providers. In Denmark, for example, designated hospitals are excluded from DRG-based payment for treating ‘complex patients’ and receive a cost-based reimbursement instead of the standard DRG-based payment. As a result, other hospitals have an incentive to transfer these patients to the designated hospitals as they would only receive the standard DRG-based payment. The Danish National Board of Health annually defines a number of requirements for hospitals eligible to treat complex patients, such as the capacity of clinical services, patient volume, experience and expertise or the access to required technical facilities.^40^ Therefore, the exclusion of highly complex patients from DRG-based payment in Denmark explicitly rewards the treatment of these patients by providers that have the necessary clinical capacity to do so.

###  Limitations

 A limitation of this study is the degree of simplification of the developed framework. This may have led to loss or misrepresentation of certain aspects of mechanisms excluded from DRG-based payments in different countries. For example, the category of excluded services/products includes a wide range of different services and products excluded in different countries, and the number of excluded services and products differs widely across countries. Similarly, the payment mechanisms for combined exclusion of special patients treated by special providers in Denmark and England are quite different, given the use of cost-based reimbursement in Denmark and top-up payments in England. However, given the complexity of payment systems, a certain degree of simplification was necessary to allow comparisons between countries. In addition, the specific features of each exclusion mechanism have been explained in more detail in the text.

 Another limitation of our research is that our approach does not allow to draw conclusions about the appropriateness of exclusion mechanisms in one country compared to another. Nor does it provide evidence on the effects of different exclusion mechanisms on quality, patient experience, or efficiency of care. Nevertheless, providing an overview about exclusion mechanisms in different countries can help researchers and policy-makers to better understand the effects of DRG-based payment systems in different countries as the available empirical and theoretical literature provides evidence on the effects of different payment mechanisms on these aspects of care.^24,32,37^

 Furthermore, individual experiences, research interests, and perceptions of experts may have influenced the choice of elements that are selected for presentation. However, we attempted to assure accuracy by collecting information through a standard questionnaire and by validating and cross-checking the information provided by national researchers.

## Conclusion

 This is the first systematic cross-country comparison of elements excluded from DRG-based payment systems and of the related additional payment components in Denmark, England, Estonia, France, Germany and the United States (Medicare). Our results show that while the complexity of exclusion mechanisms differs across countries, certain areas of care are almost always excluded from DRG-based payment (eg, certain areas of cancer care or specialized pediatrics). In addition, some countries use exclusion mechanisms to steer service provision for highly complex patients to specialized providers.

 Our results may guide policy-makers introducing or redesigning DRG-based payment systems to identify areas of care that might better be excluded from DRG-based payment. In addition, the Danish approach of incentivizing the provision of care for highly complex patients at a small number of designated hospitals can provide inspiration for policy-makers aiming to concentrate hospital care in their countries. Furthermore, researchers may benefit from applying our analytical framework to better understand the specific design features of DRG-based payment systems across countries.

## Acknowledgement

 We would like to thank Julian Pettengill who responded to the original questionnaire and provided very useful information on the United States but could not be reached to validate the information prior to submission. The paper is partially based on the Belgian Health Care Knowledge Centre (KCE) report 302 “Payment methods for hospital stays with a large variability in the care process.”

## Ethical issues

 Ethical approval was not required for this study as it did not involve primary data collection or secondary data analysis of patient data.

## Competing interests

 Authors declare that they have no competing interests.

## Authors’ contributions

 WQ, AG, and VS conceptualized and designed the study, and coordinated the acquisition of the data from all co-authors. All authors contributed to the analysis and interpretation of national information, which was then collated by VS. VS made a first draft of the manuscript, which was critically revised by all authors. All authors approved the submitted version of the manuscript.

## Funding

 The paper is based on research funded by the KCE, study no. 2014–055 (HSR). KCE contributed to the conceptualization of the study but had no role in the analysis of the data or in the drafting of the manuscript.

## Supplementary files

Supplementary file 1 contains Table S1.

Supplementary file 2. Expert Survey: Dealing With High Variability in DRG-Based Payment for Acute Care Hospitals.
