# Supplementary material for: How Denmark, England, Estonia, France, Germany, and the USA Pay for Variable, Specialized and Low Volume Care: A Cross-country Comparison of In-patient Payment Systems
Source: Int J Health Policy Manag. 2022 May 7;11(12):2940–50. doi: 10.34172/ijhpm.2022.6536 (PMC10105175; doi:10.34172/ijhpm.2022.6536)
Supplement: Supplementary file 1 — contains Table S1. [file ijhpm-11-2940-s001.pdf]

Article title: How Denmark, England, Estonia, France, Germany, and the USA Pay for Variable, Specialized and Low Volume Care: A Cross-country Comparison of In-patient Payment Systems

Journal name: International Journal of Health Policy and Management (IJHPM)

Authors' information: Wilm Quentin[1,2]*, Victor Stephani[3], Robert A. Berenson[4], Lone Bilde[5,6], Katja Grasic[7], Riina Sikkut[8], Mariama Touré[9], Alexander Geissler[10]

[1]Department of Health Care Management, Technische Universität Berlin, Berlin, Germany.

[2]European Observatory on Health Systems and Policies, Brussels, Belgium.

[3]HelloBetter, Berlin, Germany.

[4]The Urban Institute, Health Policy Center, Washington, DC, USA.

[5]Danish Institute for Applied Social Sciences Research, Copenhagen, Denmark.

[6]Danish Cancer Society Research Centre, Copenhagen, Denmark.

[7]Centre for Health Economics, University of York, York, UK.

[8]The Estonian Parliament, Tallinn, Estonia.

[9]Poverty, Health and Nutrition Division (PHND), International Food Policy Research Institute (IFPRI), Washington, DC, USA.

[10]School of Medicine, University of St. Gallen, St. Gallen, Switzerland.

(Corresponding author: wilm.quentin@tu-berlin.de)

Supplementary file 1

Table S1. Long list of countries with reasons for inclusion/exclusion

Country	Payers (multiple/single)	DRG-based payment combined with …	Reason for inclusion / exclusion
		**Included**	
**Denmark**	Common payment system (with adjustments)	Global Budgets	Recent large-scale changes in hospital-structure, systematic pathways for stroke patients
**England**	Single payer	Global budget (GB), additional payments	Transparent DRG system, no language barrier
**Estonia**	Single payer	Fee for Service (FFS) (33%), per diems (28%)	Combination of FFS and DRG-based payments
**France**	Multiple payers, common payment system	GB, additional payments	Similarity to Belgian system concerning private hospitals (e.g. FFS-payments for doctors)
**Germany**	Multiple payers, common payment system	GB, additional payments, per diems	Transparent DRG system, no language barrier, datadriven exclusion of services and patient-groups
**USA-Medicare Part A**	Multiple payer, common payment system	Depends on hospital	Separate FFS payments for physicians and FFS adjustments
		**Excluded**	
**Sweden**	Multiple payers, multiple (regional) payment systems	Differs by council	Exclusion list varies widely between counties.
**Switzerland**	Multiple payers, common payment system, negotiated prices	Additional payments	Non-transparent negotiations about prices
**Austria**	Multiple payers, common payment system	Differs by state, budgets, per diems	DRG-based payment differs by states
**Finland**	Multiple payers, multiple payment systems	Differs by district	DRG-based payment differs by hospital districts
**Israel**	Multiple payers, common payment (possibly with adjustments)	DRGs, per diems and FFS	Payment system varies by insurer
**Italy**	Single payer, common payment (with regional tariffs)	Per diems (for long stay outliers), global budget, additional payments	Very fragmented system with great variation between regions
**The Netherlands**	Multiple payers, common payment system, negotiated prices	Global budget, additional payments	DRG-like payment system is too different from other DRG-based payment systems
