# Supplementary material for: How Denmark, England, Estonia, France, Germany, and the USA Pay for Variable, Specialized and Low Volume Care: A Cross-country Comparison of In-patient Payment Systems
Source: Int J Health Policy Manag. 2022 May 7;11(12):2940–50. doi: 10.34172/ijhpm.2022.6536 (PMC10105175; doi:10.34172/ijhpm.2022.6536)
Supplement: Supplementary file 2 — Expert Survey: Dealing With High Variability in DRG-Based Payment for Acute Care Hospitals. [file ijhpm-11-2940-s002.pdf]

Article title: How Denmark, England, Estonia, France, Germany, and the USA Pay for Variable, Specialized and Low Volume Care: A Cross-country Comparison of In-patient Payment Systems

Journal name: International Journal of Health Policy and Management (IJHPM)

Authors' information: Wilm Quentin[1,2]*, Victor Stephani[3], Robert A. Berenson[4], Lone Bilde[5,6], Katja Grasic[7], Riina Sikkut[8], Mariama Touré[9], Alexander Geissler[10]

[1]Department of Health Care Management, Technische Universität Berlin, Berlin, Germany.

[2]European Observatory on Health Systems and Policies, Brussels, Belgium.

[3]HelloBetter, Berlin, Germany.

[4]The Urban Institute, Health Policy Center, Washington, DC, USA.

[5]Danish Institute for Applied Social Sciences Research, Copenhagen, Denmark.

[6]Danish Cancer Society Research Centre, Copenhagen, Denmark.

[7]Centre for Health Economics, University of York, York, UK.

[8]The Estonian Parliament, Tallinn, Estonia.

[9]Poverty, Health and Nutrition Division (PHND), International Food Policy Research Institute (IFPRI), Washington, DC, USA.

[10]School of Medicine, University of St. Gallen, St. Gallen, Switzerland.

(Corresponding author: wilm.quentin@tu-berlin.de)

Supplementary file 2. Expert Survey: Dealing With High Variability in DRGBased Payment for Acute Care Hospitals

Section 1: Background / Context of hospital payments

1) **What proportion of total (national) hospital payment is determined by DRGs?** Please fill in the table below.

Table: National hospital payments

	Local Currency	% of total acute care hospital payments
Total national hospital payment (Year)		
– Hospital payment determined by **DRGs**		
– Hospital payments for **outliers**		
– Hospital payments for excluded **patient groups (e.g. for DRGs without a cost weight)**		
– Hospital payments for excluded **services (e.g. additional FFS payments, unbundled payment)**		
– Hospital payments for excluded **departments/hospitals (e.g. ICU, palliative care)**		
– Other hospital payment mechanisms (please specify)		

2) **Development and updates: Is there a database of costs or length of stay data to inform updates of the DRG catalogue?**

   a. **What information does it include?**

   b. **How many hospitals provide data for this database?**

   c. **How long is the time-lag between data collection and use of the data for payment?**

Section 2: Exclusion from DRG-based payment system

1) The exclusion of patient groups

a. **Which patient groups are excluded from the DRG-based payment system?** (If there is a list, please provide it as an appendix.)

b. **Determining the list of excluded patient groups :**

   i. Responsibilities: Which institution is responsible? Which actors are involved in the process?

   ii. Process: Is the list of excluded patient groups regularly revised (how often)? Or was it defined only once (and how)? Is there a formal process for updating the list? For example, can medical specialties apply/suggest patient groups to be excluded? Who takes the final decision and how?

   iii. Empirical basis: Is the list of excluded patient groups defined on an empirical basis? For example, is there a (cost or length of stay) data base, where patients with highly variable costs are identified. If this is the case, please explain also the rules that are used to identify patient groups that are to be excluded? (For example, a rule could be that patient groups for which the standard deviation is larger than the mean of costs are to be excluded.)

c. **What is the percentage of all patients/cases that is excluded?** Please provide the total number of hospital cases (in year xxxx) and the number of cases for which hospitals were not paid on the basis of DRGs.

d. **How are hospitals paid for these patients?** Please explain:

   i. The payment system, i.e. whether hospitals receive a budget/fee-for-service payments or per diems;

   ii. How the size of the payment is determined, e.g. based on historic costs of individual providers or average costs across similar providers;

   iii. The scope of the payment, i.e. what services are covered by the payment.

2) The exclusion of services and products

a. **Which products or services are excluded?** (If there is a list of excluded products/services, please provide it as an appendix.)

b. **Determining the products/services that are to be excluded**

   i. Responsibilities: Which institution is responsible? Which actors are involved in the process?

   ii. Process: Is the list of excluded services/products regularly revised (how often)? Or was it defined only once (and how)? Is there a formal process for updating the list? For example, can medical specialties apply/suggest that certain procedures are to be excluded? Who takes the final decision and how?

   iii. Empirical basis: Is the list of excluded services/products defined on an empirical basis? For example, is there a (cost or length of stay) data base, where high cost services/products are identified. If this is the case, please explain also the rules that are used to identify services/products that are to be excluded? (For example, a rule could be that services, which account for more than half of the costs of an inpatient stay and which are provided as part of at least three different DRGs are to be excluded.)

c. **How are hospitals paid for these products/services? Please explain**

   i. The payment system, i.e. whether hospitals receive a budget/fee-for-service payments or per diems

   ii. How the size of the payment is determined, e.g. based on historic costs of individual providers or average costs across similar providers?

3) **The exclusion of certain hospitals or hospital departments**

a. **Which hospitals or departments are excluded?** (If there is a list of excluded hospitals and/or hospital departments please provide it as an appendix.)

b. **Determining the list of hospitals/departments that are to be excluded**

   i. Responsibilities: Which institution is responsible for determining excluded hospitals / departments? What actors are involved?

   ii. Process: Is the list of excluded hospitals / departments regularly revised (how often)? Or was it defined only once (and how)? Is there a formal process for updating the list? For example, can medical specialties apply/suggest that departments are to be excluded? Who takes the final decision and how?

   iii. Empirical basis: Is the list of excluded hospitals / departments defined on an empirical basis? For example, is there a data base, where cost data from hospitals / departments shows that cost are high variable? If this is the case, please explain also the rules that are used to identify hospitals / departments that are to be excluded? (For example, a rule could be that hospital departments for which the standard deviation is larger than the mean of costs are to be excluded.)

c. **What is the percentage of excluded hospitals/departments?** Please provide the total number of hospitals (in year xxxx) and the number of hospitals that were not paid on the basis of DRGs.

d. **How are excluded hospitals/departments paid?**

   i. The payment system, i.e. whether hospitals receive a budget/fee-for-service payments or per diems;

   ii. How the size of the payment is determined, e.g. based on historic costs of individual departments or average costs across similar departments;

4) Outliers

a. **How are outliers defined?** (based on which data)

b. **Which institution is responsible for determining the outlier definition?**

c. **What payment mechanisms exist to take outliers into account?**

d. **How are outliers paid?**

5) Other

a. **Are there other mechanisms used to pay for highly complex or specialised care outside the DRG-based payment system?**

b. **If yes, what is excluded?**

Section 3. Main challenges, debates and reforms

Please focus on challenges and debates related to the problem of patients with high variability of costs (if this is an issue in your country).

1) **Main Challenges**

    a. **What are the main challenges for the hospital payment system?**

2) **Debates**

    a. **Are there any current debates about the DRG-based payment system?**

    b. **What are the debates about?** In particular, we would be interested to know if the payment of hospitals for highly complex/highly variable patients has been an issue.

    c. **Who is taking part in the debates?**

3) **Reforms**

    a. **Have there been recent reforms of the DRG-based hospital payment system?**

    b. **Have there been reforms introducing standardised care pathways involving multiple hospitals? If yes, how are these reflected by the hospital payment system?** For example, stroke patients may be systematically transferred from an initial hospital to a more specialised hospital (or vice versa), and there may be rules for splitting the payment.

    c. **Have there been evaluation studies on the impact of reforms that excluded patients/services/hospitals from the DRG-payment system?** (E.g. is there a bias towards/against smaller hospitals?)
